# Ilizarov Method for the Management of Infected Nonunion of the Femur With or Without Bony Defects

**DOI:** 10.7759/cureus.105914

**Published:** 2026-03-26

**Authors:** Nitya Ranjan Balo, Md. Abdul Gani Mollah, Nazmul Huda Shetu, Md. Syedur Rahaman, ASM Nurul Alam Siddique, BM Rahgir Mahmud

**Affiliations:** 1 Orthopaedics and Traumatology, National Institute of Traumatology and Orthopaedic Rehabilitation (NITOR), Dhaka, BGD

**Keywords:** asami criteria, bone defect, distraction osteogenesis, femur, ilizarov external fixator, infection, nonunion

## Abstract

Background

Infected nonunion of the femur is a complex and disabling condition characterized by persistent infection, bone loss, limb length discrepancy, deformity, and joint stiffness. Management is particularly challenging in patients with multiple previous failed surgeries. The Ilizarov external fixator, based on the principles of distraction osteogenesis, offers a biological and mechanical approach that enables stable fixation, infection control, and simultaneous bone reconstruction.

Methods

This retrospective observational study was conducted at the National Institute of Traumatology and Orthopaedic Rehabilitation (NITOR), Dhaka, Bangladesh, from July 2019 to July 2024. A total of 34 patients (aged 16-65 years) with infected nonunion of the femur, with or without bone defects, were included. Diagnosis was established based on a combination of clinical (persistent pain, sinus tract, purulent discharge, local inflammatory signs, abnormal mobility), radiological (sequestrum, bone lysis, periosteal reaction, implant loosening), laboratory (elevated erythrocyte sedimentation rate (ESR), C-reactive protein (CRP), leukocytosis), and microbiological findings where available. Radiological nonunion was defined as the absence of healing for at least 6-9 months with no progression over three months.

All patients underwent thorough surgical debridement followed by stabilization using the Ilizarov external fixator, with or without corticotomy. Segmental bone transport was performed for bone defects greater than 1.5 cm. Postoperative management included early mobilization, physiotherapy, and culture-guided antibiotic therapy. Outcomes were assessed using the Association for the Study and Application of the Method of Ilizarov (ASAMI) criteria for bone and functional results, and complications were classified according to Paley's classification.

Results

The mean age of the patients was 36.82±13.48 years, with a predominance of males (91.2%). The mean follow-up duration was 17.79±5.85 months. Bone defects were present in 85.3% of cases, with a mean defect size of 2.84±1.81 cm and a mean limb length discrepancy of 3.85±1.74 cm. Segmental bone transport was performed in 16 patients, achieving a mean regenerate length of 5 cm and a mean lengthening index of 2.47±0.36 cm/month. The mean duration of external fixation was 11.91±2.41 months.

Infection control was achieved in all patients based on clinical and laboratory parameters during follow-up. According to the ASAMI criteria, bone results were excellent or good in 94.1% of patients, while functional results were excellent or good in 85.3%. Common complications included pin tract infection, knee stiffness, wire breakage, and docking site nonunion.

Conclusion

This study suggests that the Ilizarov external fixator is associated with favorable outcomes in the management of infected nonunion of the femur, including high rates of bone union and infection control. However, given the retrospective design and absence of a comparison group, these findings should be interpreted with caution. Functional outcomes may be limited by complications such as joint stiffness, highlighting the need for comprehensive postoperative rehabilitation. Further prospective and comparative studies are required to validate these results.

## Introduction

Infection of bones and joints has a detrimental effect on the overall health of the patient. Post-traumatic open fracture is the commonest cause of long bone infection due to the direct contamination of organisms. Though *Staphylococcus aureus *is the main causative organism, co-infection with multiple organisms is terrifyingly increasing day by day, posing an additional challenge. Management of infected nonunion of long bones remains challenging because microorganisms form biofilms and adhere to bone and implants, significantly reducing antibiotic penetration and treatment efficacy [1,2]. 

Host factors are important indicators of the predictable outcome of the disease. Normal hosts have better outcomes compared to immunocompromised patients who are suffering from diabetes mellitus, peripheral vascular disease, sensory neuropathy, and malignancy [3]. Host immune response is also impaired in the presence of dead and devitalized tissue within the infective foci of the bones [4].

Infected nonunion is defined as failure of fracture healing beyond the expected period, typically 6-8 months, in the presence of persistent bone infection. It usually occurs as a consequence of trauma or surgery [5].

Apart from infection and nonunion, other drawbacks of infected nonunion of the femur include bony defect, multiple discharging sinuses, regional osteoporosis, limb length discrepancy, deformity, knee joint stiffness, soft tissue fibrosis, and re-fracture [6-8].

The goals of treatment are to eradicate infection, unite the bone, address the drawbacks (deformity, limb length discrepancy, and soft tissue problems), and, in the end, obtain a functional limb [9].

Traditional treatment options for infected nonunion are radical debridement, internal fixation with bone grafting, and a period of antibiotic therapy. The Ilizarov external fixator is unique for its universal mechanism and principles, which are based on distraction osteogenesis, whereby controlled mechanical tension stimulates the biological regeneration of bones and surrounding soft tissues. It provides transaxial stability of the bones and favors micromotion at the nonunion site, which augments bone healing and early mobilization. On the other hand, rapid regeneration induced by corticotomy helps burn out the infection. Not only does it unite the bones and eradicate the infection, but it also addresses all the drawbacks associated with infected nonunion simultaneously [10].

This study aimed to evaluate the clinical outcomes of the Ilizarov method in the management of infected nonunion of the femur with or without bony defects, with particular reference to bone union, infection eradication, functional recovery, limb length discrepancy, and complication rates, assessed using the Association for the Study and Application of the Method of Ilizarov (ASAMI) criteria.

## Materials and methods

This was an observational descriptive retrospective study, corresponding to level IV evidence. It was conducted at the National Institute of Traumatology and Orthopaedic Rehabilitation (NITOR) in Dhaka, Bangladesh, from July 2019 to July 2024. A formal institutional review board approval was obtained from the institute's Institutional Review Board (approval number: NITOR/ACADEMY/2019/22) prior to commencement of the study.

Diagnosis of infected nonunion was established based on a combination of clinical (persistent pain, sinus tract, purulent discharge, local inflammatory signs, abnormal mobility), radiological (sequestrum, bone lysis, periosteal reaction, implant loosening), laboratory (elevated erythrocyte sedimentation rate (ESR), C-reactive protein (CRP), leukocytosis), and microbiological findings where available. Inclusion criteria of this study were ambulatory patients, aged between 15 and 65 years, both male and female, with established infected nonunion of the femur following trauma or previous surgical interventions. Exclusion criteria include nonunion due to pathological fracture, aseptic nonunion, nonunion involving articular parts or joints, and patients with a psychological disorder or polytrauma. In total, 34 patients were enrolled from the outpatient department after strictly considering the inclusion and exclusion criteria consecutively.

After counseling for surgical technique, informed written consent was taken from each of the patients. Preoperative and postoperative data were recorded. Data which were collected from medical records were reviewed and analyzed using descriptive statistical methods. Continuous variables like age, bone defect size, limb length discrepancy, duration of external fixation time, lengthening index, and follow-up duration were expressed as mean±standard deviation with range. Categorical variables like sex and treatment outcomes were presented as percentages. Treatment outcomes were evaluated using standard outcome assessment criteria. No comparative or inferential statistical tests were applied due to the descriptive nature of the study. Statistical analysis was performed using IBM SPSS Statistics for Windows, Version 31.0 (IBM Corp., Armonk, New York, United States). 

Surgical technique

With all aseptic precautions, all patients underwent surgery under spinal anesthesia. Deep-seated pus was taken and sent for culture and sensitivity test. Dead and devitalized tissues as well as implants if present were removed from the nonunion site. Surgical debridement was continued until punctate bleeding came from the bone ends. Then the bone was stabilized using the Ilizarov external fixator [10].

Acute docking of bone ends was done when the gap was less than 5 cm, and if the gap was more than 5 cm, then ascending or descending corticotomy was done as per the site of nonunion. Following a latency period of 5-7 days after corticotomy, distraction was initiated at a standard rate of 1 mm/day, divided into four increments for bone segment transport. Active movement and weight-bearing started as early as possible depending on the pain permissibility of the patient. Movement of the joints and physiotherapy were also started gradually. Pin sites were cleansed with chlorhexidine solution and secured with a sterile gauge, keeping them under a rubber stopper. Postoperatively, antibiotics were continued for six weeks based on the culture sensitivity report [10-12]. 

Patients were followed up every 2-4 weeks during the distraction phase and monthly thereafter until union. Clinical and radiological assessments were performed at each visit. After fixator removal, follow-up continued at regular intervals to monitor consolidation and functional recovery.

## Results

A total of 34 patients who underwent treatment with the Ilizarov method and completed follow-up were included in this study. This study was not designed to perform a subgroup comparison between patients with and without bone defects.

The mean age of the patients was 36.82 years (SD 13.48; range 16-65 years). There were 31 males (91.2%) and three females (8.8%). The mean duration of follow-up was 17.79 months (SD 5.85; range 9-31 months). Out of 34 patients, 29 (85.3%) had associated bony defects. The mean bone defect size was 2.84 cm (SD 1.81; range 1-9 cm). The mean limb length discrepancy was 3.85 cm (SD 1.74; range 2-8 cm). A total of 16 patients underwent segmental bone transport. The mean length of regenerated bone was 5 cm (range 2-9 cm). The mean lengthening index was 2.47 cm/month (SD 0.364; range 2-3 cm/month). The mean duration of treatment, defined as the time to removal of the Ilizarov external fixator, was 11.91 months (SD 2.41; range 7-19 months). All but one patient had undergone at least one previous surgical procedure prior to Ilizarov application. The mean number of previous surgeries was 3.33 (SD 1.08; range 1-5). Infection eradication was assessed using predefined clinical, laboratory, radiological, and follow-up criteria. Clinical criteria included complete healing of the sinus tract, absence of discharge, and no local signs of infection. Laboratory criteria included the normalization of ESR and CRP. Radiological criteria included the absence of osteolysis or sequestrum formation. Follow-up criteria included no recurrence of infection during the follow-up period. Based on these criteria, infection was successfully eradicated in all patients, resulting in a 100% infection cure rate in this retrospective study.

Outcome was assessed based on bone and functional results as per the ASAMI scoring criteria [13]. Bone result was excellent in 26 patients, good in six patients, and fair in two patients. Functional result was excellent in nine patients, good in 20 patients, and fair in five patients. There was no poor result. Bone results and functional results as per the ASAMI scoring criteria are shown in Tables 1-2 [13].

**Table 1:** Bone results as per the ASAMI scoring criteria (n=34) ASAMI: Association for the Study and Application of the Method of Ilizarov

Bone results	Number of patients	Percentages
Excellent	26	76.47%
Good	6	17.64%
Fair	2	5.88%
Poor	0	0%

**Table 2:** Functional results as per the ASAMI scoring criteria (n=34) ASAMI: Association for the Study and Application of the Method of Ilizarov

Functional results	Number of patients	Percentages
Excellent	9	26.47%
Good	20	58.82%
Fair	5	14.7%
Poor	0	0%

Complications occurred during treatment, entitled as problems, obstacles, and true complications based on Paley's working classification [13]. Complications are listed in Table 3.

**Table 3:** Problems, obstacles, and true complications based on Paley's working classification

Complications	Category of complications	Number of patients
Problems	Pin tract infection	14
	Pain	16
	Delayed union	3
	Axis deviation	4
Obstacles	Wire breakage	9
	Premature consolidation	1
	Interposition of the soft tissue	6
True complications	Ankle edema	10
	Depression	18
	Knee stiffness	22
	Axis deviation	2

During bone segment transport, six patients developed axis deviation, which was corrected during the course of treatment, but two had permanent deformity at the end of treatment. Six patients had docking site nonunion, all of whom underwent surgical intervention for the clearance of interposing soft tissue. The accordion technique was needed for a few patients who had poor-quality regeneration in the corticotomy site [14]. Pin tract infection was noticed in 14 patients and was managed by proper wound site care and local antibiotics. Nine patients had broken Ilizarov wires, which were replaced under local anesthesia. Knee stiffness was present in 22 patients during their enrolment, of whom seven underwent quadricepsplasty at the end of treatment but no one achieved a full range of knee movement. No patient had complex regional pain syndrome, but 10 patients had persistent ankle and leg edema.

**Figure 1:**
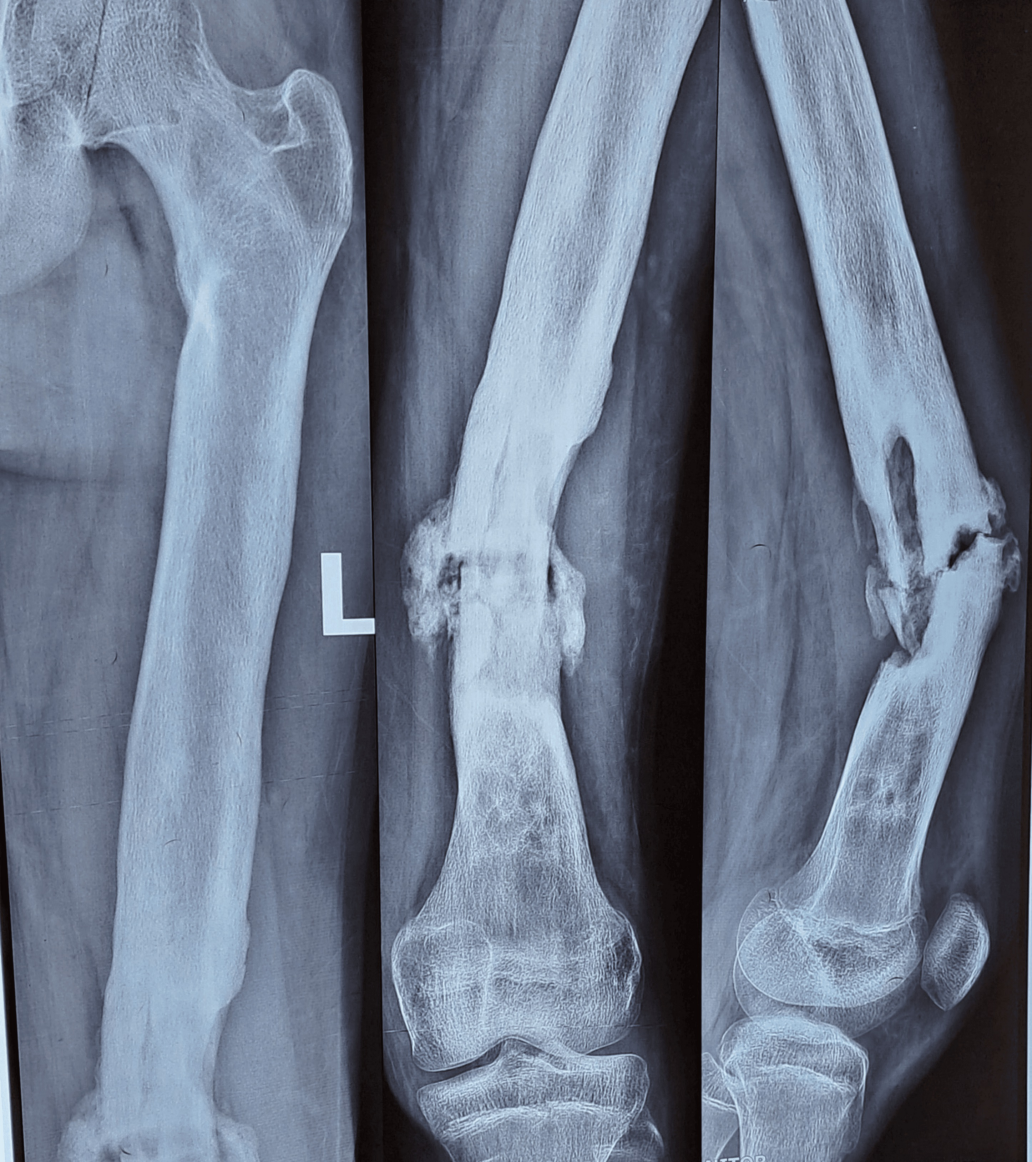
Preoperative X-ray of an infected nonunion of the femur with a bony defect

**Figure 2:**
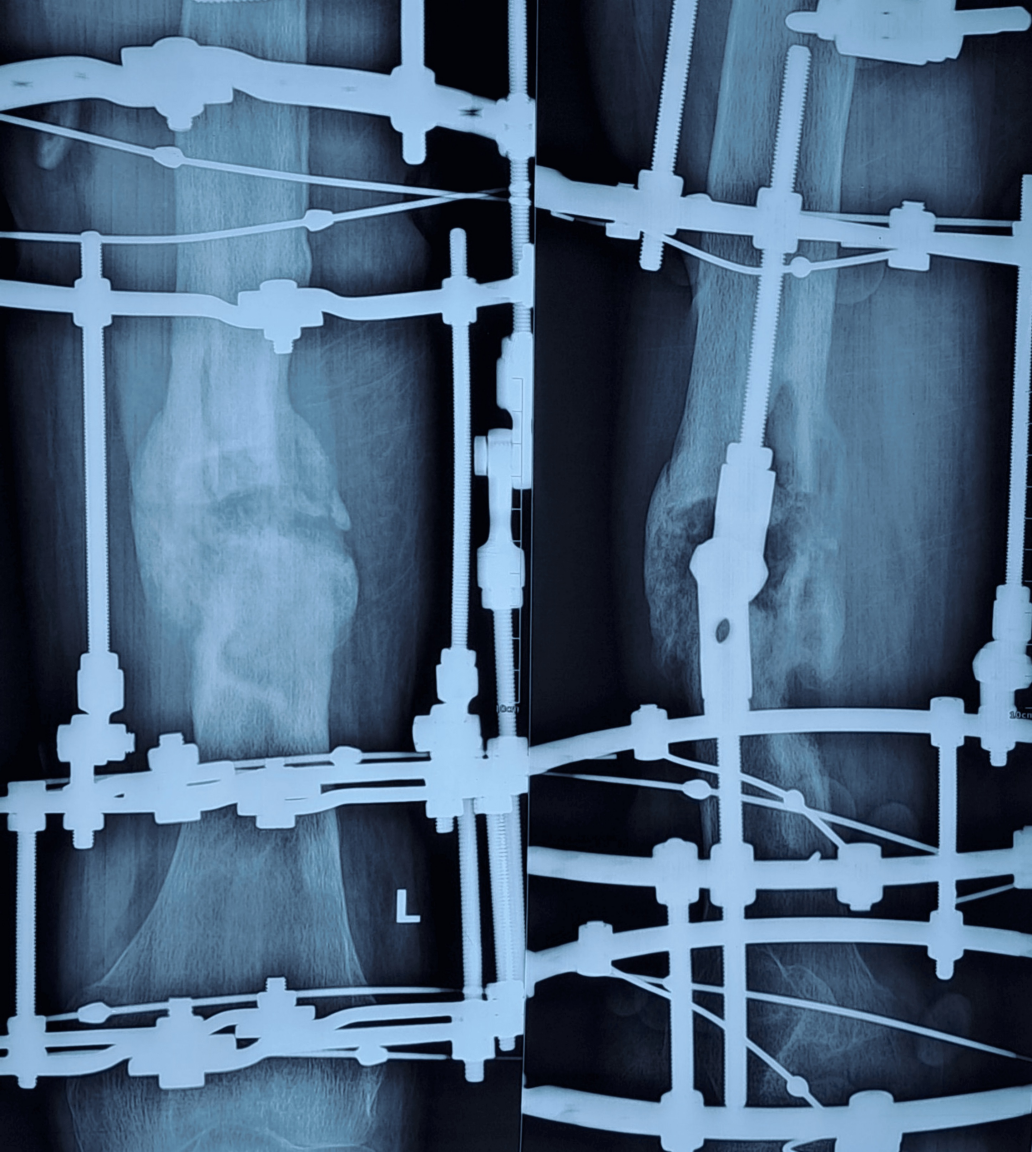
Postoperative X-ray with an Ilizarov external fixator

**Figure 3:**
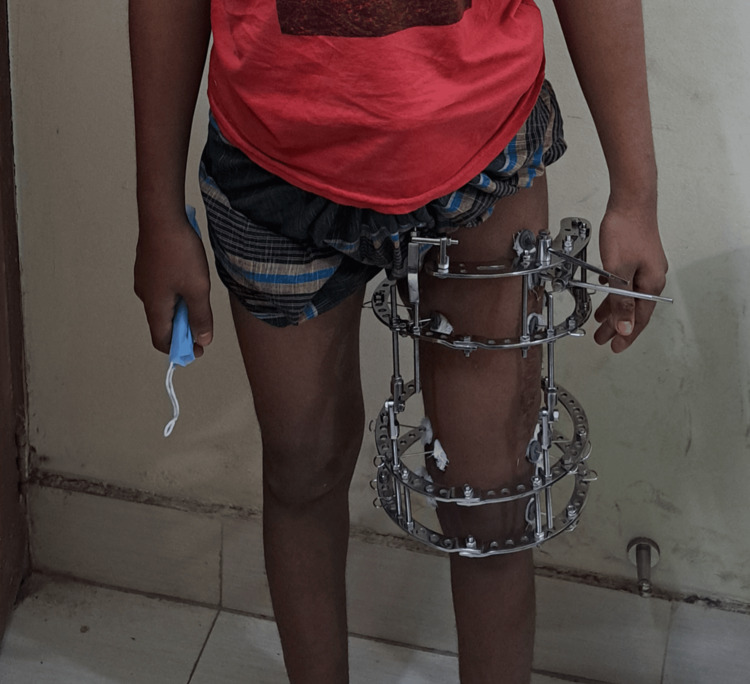
Patient with an Ilizarov external fixator

**Figure 4:**
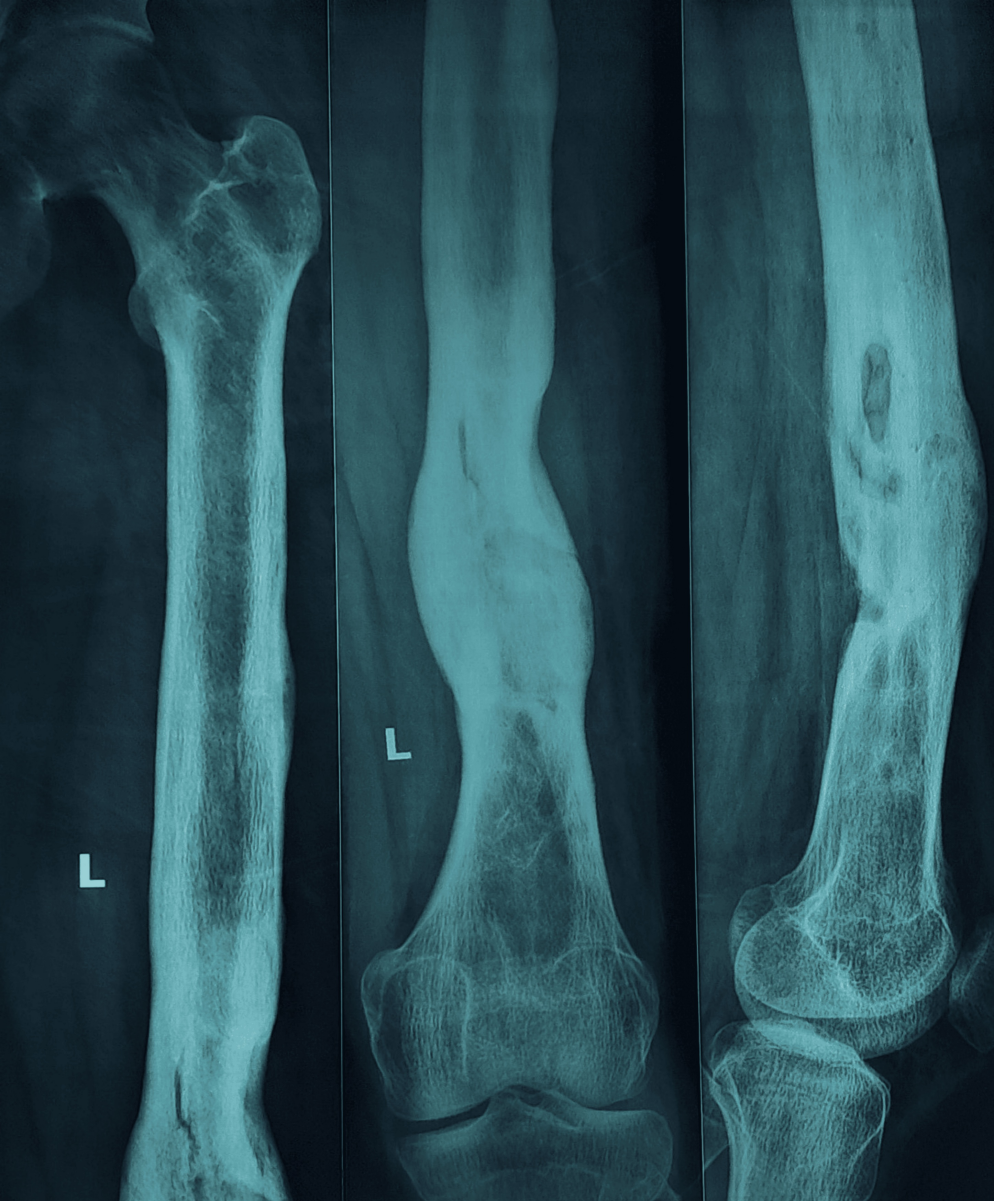
United, healthy, and well-consolidated femur after the removal of the Ilizarov external fixator

Figures 1-4 are case illustrations showing a preoperative X-ray of an infected nonunion of the femur with a bony defect, a postoperative X-ray with an Ilizarov external fixator, a patient with an Ilizarov external fixator, and a united, healthy, and well-consolidated femur after the removal of the Ilizarov external fixator.

## Discussion

Almost all patients of this retrospective study presented not only infection and nonunion but also one or more other morbidities, such as bony defect, deformity, limb length discrepancy, and stiffness. These problems are also highlighted in other studies [6,10,11].

In this descriptive study, 34 patients with infected nonunion of the femur were evaluated following treatment under the principle of the Ilizarov method. The mean age was 36.82 years, with a marked male predominance (91.2%), comparable with previous reports, where young adult males constitute the majority due to high-energy trauma and occupational exposure [15,16]. The fact that almost all patients underwent multiple previous surgical procedures (mean 3.33 operations) highlights the complexity of infected femoral nonunion and the failure of conventional internal fixation methods, as also reported in other series [17,18].

The presence of bone defects in 85.3% of patients and a mean limb length discrepancy of 3.85 cm reflects the severe nature of infection-related bone loss. Similar magnitudes of bone defects and limb shortening have been reported in studies dealing with post-infective nonunion of long bones managed by distraction osteogenesis [19,20]. One of the most significant findings of this study is the 100% infection eradication rate. This confirms the biological advantage of the Ilizarov technique, which allows radical debridement, stable fixation, improved local vascularity, and avoidance of retained internal implants in an infected field. Several authors have reported infection control rates ranging from 90% to 100% using circular external fixation in infected femoral nonunion, supporting the reliability of this method [10,12,21]. Segmental bone transport was required in 16 patients, with a mean regenerate length of 5 cm and a mean lengthening index of 2.47 cm/month. These findings are consistent with Ilizarov principles and comparable to previously published series, where lengthening indices ranged between 1.5 and 3 cm/month depending on defect size, patient compliance, and regenerate quality [12,22]. The need for the accordion technique in three patients due to poor regenerate formation has also been described by Paley and others as an effective biological stimulus to improve osteogenesis in compromised regenerate bone [14,23]. Bone results in this study were excellent or good in 94.1% of patients, while functional outcomes were excellent or good in 85.3% of cases. These results are comparable to other series demonstrating excellent-to-good bone results in 85-95% of patients treated with Ilizarov fixation for infected femoral nonunion [10,13,16,24]. The observed disparity between favorable bone outcomes and comparatively lower functional results is well-recognized in Ilizarov literature and is largely attributable to joint stiffness and muscle fibrosis rather than failure of bone union [7,25].

Axis deviation during bone transport occurred in six patients, a well-recognized complication of distraction osteogenesis, which was mostly correctable during treatment. Docking site nonunion occurred in six patients and required surgical intervention, a finding consistent with previous studies that emphasize the need for meticulous docking site management, including compression and bone grafting when necessary [10,26]. Pin tract infection was the most frequent complication, observed in 41.2% of patients, but was successfully managed with local care and antibiotics. This incidence falls within the widely reported range of 30-70% in circular external fixation [27,28]. Knee stiffness remained a major functional limitation despite quadricepsplasty in some patients, reinforcing the importance of aggressive physiotherapy and early joint mobilization, as emphasized in earlier studies [29]. Wire loosening or breakage was managed with timely replacement or adjustment. These complications, although frequent, were generally manageable and did not significantly affect the final outcomes.

A notable finding in our study was the high prevalence of depression (52.9%) during treatment. This likely reflects the prolonged duration of external fixation, physical discomfort, and functional limitations associated with the Ilizarov method. Psychological distress may adversely affect rehabilitation and overall functional outcomes. This aspect is underreported in many studies and highlights the importance of incorporating psychological support as part of comprehensive patient care.

The limitations of this study include its retrospective design, its potential for selection and information bias, its relatively small sample size, its lack of a control group treated with alternative fixation methods, and its single-center nature, which may limit generalizability. Additionally, functional outcomes may have been influenced by patient compliance and rehabilitation variability. Despite these limitations, the study provides valuable evidence supporting the role of Ilizarov external fixation in complex infected femoral nonunion.

## Conclusions

Infected nonunion of the femur represents a complex reconstructive problem for the orthopedic surgeon. Prolonged application of the Ilizarov external fixator around the thigh is often uncomfortable for the patients and may impose psychological stress during the course of treatment. Nevertheless, the biological and mechanical principles of the Ilizarov technique, particularly gradual load transmission and controlled stress, facilitate the simultaneous eradication of infection, the restoration of bone continuity, and limb salvage.

This study suggests that the Ilizarov external fixator is associated with favorable outcomes in the management of infected nonunion of the femur, including high rates of bone union and infection control. However, given the retrospective design and absence of a comparison group, these findings should be interpreted with caution. Functional outcomes may be limited by complications such as joint stiffness, highlighting the need for comprehensive postoperative rehabilitation. Further prospective and comparative studies are required to validate these results.
